# The Potential of MicroRNAs as Novel Biomarkers for Transplant Rejection

**DOI:** 10.1155/2017/4072364

**Published:** 2017-01-16

**Authors:** Matthias Hamdorf, Satoru Kawakita, Matthew Everly

**Affiliations:** Terasaki Foundation Laboratory, 11570 W. Olympic Blvd., Los Angeles, CA 90064, USA

## Abstract

The control of gene expression by microRNAs (miRNAs, miR) influences many cellular functions, including cellular differentiation, cell proliferation, cell development, and functional regulation of the immune system. Recently, miRNAs have been detected in serum, plasma, and urine and circulating miR profiles have been associated with a variety of diseases. Rejection is one of the major causes of allograft failure and preventing and treating acute rejection are the central task for clinicians working with transplant patients. Invasive biopsies used in monitoring rejection are burdensome and risky to transplant patients. Novel and easily accessible biomarkers of acute rejection could make it possible to detect rejection earlier and make more fine-tuned calibration of immunosuppressive or new target treatment possible. In this review, we discuss whether circulating miRNA can serve as an early noninvasive diagnostic biomarker and an expression fingerprint of allograft rejection and transplant failure. Understanding the regulatory interplay of relevant miRNAs and the rejecting allograft will result in a better understanding of the molecular pathophysiology of alloimmune injury.

## 1. Introduction

**Figure 1:**
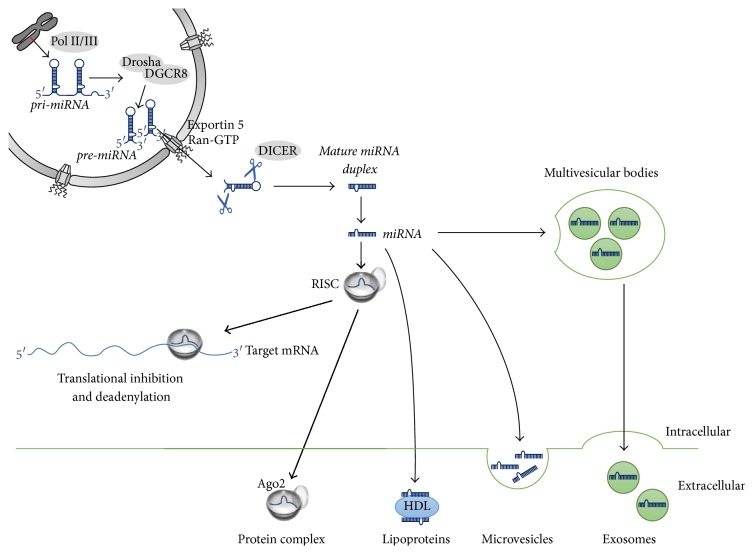
Biogenesis and release of miRNAs. Pri-miRNAs are transcribed in the nucleus by RNA polymerase II/III and processed by the ribonuclease Drosha into hairpin RNAs (pre-miRNA). The stem loops are exported into the cytoplasm using Exportin 5 and Ran-GTP and further cleaved by Dicer to yield 21–23 nucleotide duplexes. The duplexes are unwound and can be loaded directly into the RISC complex and guide translational repression of target mRNAs or they can be released from the cells in protein complexes, bound to lipoproteins, packed in microvesicles, or secreted in exosomes.

MicroRNAMicroRNAs (miRNAs, miRs) are a class of small (~22 nt) noncoding molecules that inhibit translational initiation and stimulate decay of mRNA targets [1, 2]. MiRs are transcribed by RNA polymerase II/III and processed by the RNAse III enzymes Drosha and its binding partner DGCR8 in the nucleus and Dicer in the cytoplasm, to produce short double-stranded RNAs. One strand of the double-stranded RNA is loaded into the Argonaute (Ago) protein and forms the miR-mediated silencing complex (miRISC). MiRs guide miRISC to pair with imperfect complementarity to sequences in target mRNAs, resulting in their subsequent destabilization and translational repression [3]. The target mRNA recognition by the miRISC is mediated by the “seed sequence” nucleotide 2 to 8 [4, 5]. Recent data show that 35–40% of miR binding sites are found in the 3′UTRs, 40–50% in coding regions, and <5% in the 5′UTR mRNA regions [6, 7]. Greater than 60% of the human transcriptome is predicted to be under miR regulation, making this posttranscriptional control pathway as important as proteins in the regulation of cell functions [2]. It is clear that miRs play vital roles in regulating diverse functions in normal and diseased cells [8, 9]. Recent studies have shown that in addition to intracellular regulatory functions, miRs can be secreted and detected in bodily fluids such as blood and urine. The secreted miRs are associated with proteins (Ago2), lipoprotein complexes, or packaged into microvesicles like exosomes. Circulating miRNAs are very stable and resistant to treatment with ribonucleases, freezing/thawing cycles, and other drastic experimental conditions [10]. Several studies have shown that secreted miRNAs can function as a second messenger. MiRNAs packed into exosomes or HDL can be taken up as an active component by neighboring cells and induce cell modification/regulation [10, 11]. The biogenesis, function, and export of miRNAs are shown in Figure 1. Recent reports have gone even further by reporting a much more complex picture of the strong regulatory functions of a diversity of other small ncRNA species such as piwi-interacting RNAs (piRNA) or long noncoding RNAs (lncRNA). PiRNA were identified in germline cells as regulators of transposons. They depend on a specific PIWI clade protein and their biogenesis is independent of Dicer [12, 13]. LncRNAs are a large and diverse class of transcribed RNA molecules with a length of more than 200 nucleotides that do not encode proteins. To date, very few lncRNAs have been characterized in detail. However, it is clear that lncRNAs are important regulators of gene expression and are thought to have a wide range of functions in cellular and developmental processes [14, 15]. A short overview of selected RNA species and their functions is shown in Table 1. In summary, the identification of miRNAs and small RNA species seems to represent only the tip of the iceberg and the prediction of an individual miRNA; its target and function in health and disease are one of the big challenges in research.

## 2. Use of miRNAs in Transplantation

The few publications that exist on the topic of miRNAs and transplants focus on miRNAs isolated from biopsies. An overview of the different studies is given in Table 2. This review focuses on a short overview of the transplanted organ, the identified miRNA pattern, and shared common pathways.

### 2.1. miRNAs and Pathways Involved in Transplant Rejection

#### 2.1.1. TGF-Beta Signaling Pathway

An inflammatory reaction takes place during the acute or chronic rejection of an organ. Among different cytokines such as IL-6 [38] and inflammatory mediators elicited during inflammation, TGF-*β*1 is considered the main mediator and inducer of fibrosis [39]. TGF-*β*1 belongs to a family of growth factors that includes TGF-*β*s, activins, and bone morphogenic proteins (BMPs). TGF-*β*1 and BMP-7 are key determinant factors in peritoneal cell plasticity and in particular, the predominance of one or the other may determine the epithelial or mesenchymal phenotype [40, 41]. Among the different organs and studies, the TGF-beta signaling pathway is the predominant pathway for organ rejection. Wilfingseder et al. (kidney) [42], Xu et al. (lung) [21], and Joshi et al. (liver) [18] described in their studies that miR-548d, miRNA-203, and miRNA-146a are regulators of SMAD4. After activation, SMAD4 forms homomeric and heteromeric complexes and translocates to the nucleus to initiate TGF-beta induced transcription. The downregulation of the miRNAs during rejection leads to an overexpression of SMAD4 and enhanced signaling. The receptor of TGF-beta (TGF*β*R2) is regulated by miR-548d and miRNA-200a and miRNA-141 and downregulation leads to a higher density of the receptor on the cell surface. The miRNA-200 family is described in the literature as involved in inflammation and IL-6 regulation. In an additional publication, Xu et al. defined the role of miRNA-144 in fibroproliferation leading to bronchiolitis obliterans syndrome (BOS) [20]. MiRNA-144 is strongly upregulated in BOS patients and downregulates TGIF1, a suppressor of TGF-beta induced transcription. The absence of the suppressor leads to an elevated production level of FGF, TGF-beta, and VEGF and promotes the onset of fibroses and organ rejection [20]. Discrimination of cases with fibrosis by miRNA profiles is certainly of interest in acute rejection as well as chronic rejection. The microRNA let-7c targets the TGF-beta and the WNT1 signaling pathway and can enhance the function of the other miRs [43]. In mouse transplantation models, it has been shown that miR-21 is one of the driving agents of fibrosis in acute cardiac allograft rejection [44]. In general, all miRs that are able to promote TGF-beta signaling and the development of fibrosis are drivers of rejection. The aforementioned publications lay out the mechanisms of miRNA and their function in the TGF-beta signaling pathways as a model for the interplay and modulation coursed by miRNAs.

#### 2.1.2. T-Cell, B-Cell, and Macrophage Signaling and Development

**Figure 2:**
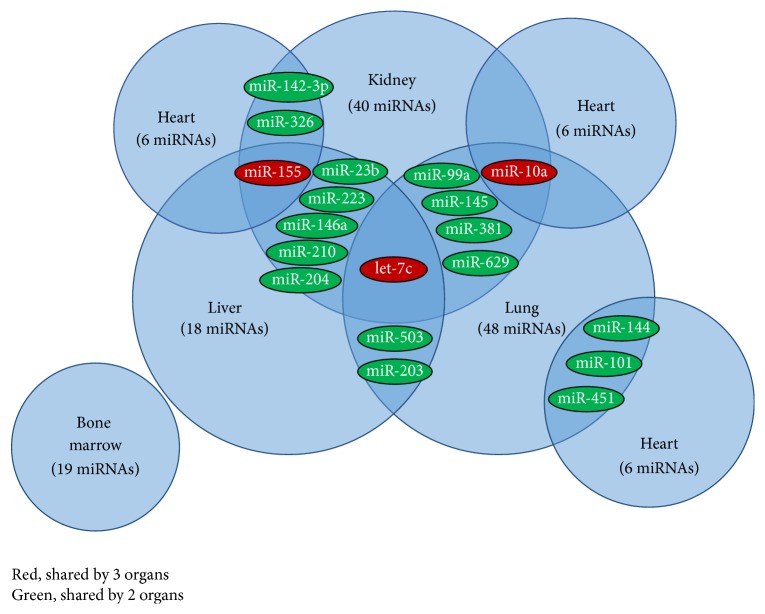
Comparison of overlapping miRs in the different studies. Color-labeled miRs indicate a miRNA shared by 2 (green) and 3 (red) organs, respectively. The number in parentheses represents nonoverlapping miRNAs detected for the corresponding organ.

Wilflingseder et al. showed that miR-182-5p expression is profoundly correlated with kidney tissue injury. MiR-182-5p can be activated by IL-2 and STAT5 and act as an inhibitor of FOXO1 expression. FOXO1 acts as a master cellular regulator of a variety of cellular processes including cell survival, apoptosis, proliferation, and metabolism. FOXO1 also plays a critical role in the homeostasis of cells of the immune system including T-cells, B-cells, and neutrophils [31]. Further, the absence of FOXO1 was shown to severely reduce the development of FOXP3+ regulatory T-cells (T_regs_). Those T_regs_ that developed were found to be nonfunctional in vivo and downregulation of FOXO1 in T-cells resulted in lymphocyte infiltration and promotion of inflammation and immune response induced rejection of the transplant [45]. In addition, Wei et al. observed in their study that mice with rejecting cardiac allografts had significantly higher levels of miR-182 in the grafts, infiltrated mononuclear cells, and plasma compared to syngeneic transplants. These findings further support the potential of miRNAs as a biomarker for organ rejection [46]. One miRNA that has an overlap in three of the analyzed organs (see Figure 2) is let-7c. In the literature, let-7c is discussed as an important regulator of HSCs by controlling signaling of TGF-beta and WNT1 and plays a role in the development of T_H_1 cells [43]. Other publications show that the polarization and activation of macrophages are dependent on let-7c [47, 48]. The development of T_H_1 cells in addition to the regulation of macrophages may play an important role in the recognition of the transplant and result in its rejection. Additionally, let-7c plays an important role in cardiomyogenesis [49] and may interfere with the replacement of tissue that is damaged by the immune system in heart transplants. Surprisingly, none of the cardiac tissue relevant miRs (miR-1, miR-133a, miR-208a/b, and miR-499) [50] are found to be affected by acute rejection. In particular for cardioprotection, a recent review discusses the importance of exosomes during normal health and injury and the interaction with the immune system [51].

Xu et al. found four differentially expressed miRNAs (miR-133b, miR-134, miR-433, and miR-628-5p) in the lungs that modulate the B-cell signaling pathway in different stages and regulate autoimmunity, immunoglobulin (Ig) production, and immune response. The study showed a significant downregulation of these miRNAs in donor specific antibody (DSA) positive BOS positive patients. The study argues that the downregulation leads to overactivation of the B-cell mediated immune response and to the DSA-induced rejection of the transplant [21].

### 2.2. miRNAs as Potential Noninvasive Biomarkers for Acute and Chronic Rejection

*Liver*. In many studies, miRNA-122 was discovered in mice and humans as a very liver-specific miRNA. In liver patients, it was demonstrated that hepatocyte-derived miRNAs miR-122, miR-148a, and miR-194 correlated with hepatic injury and acute rejection after liver transplantation [16]. The serum level of these miRNAs was significantly increased in patients with liver injury induced by rejection and there was a strongly positive correlation with the clinically used aminotransferase diagnostic marker. Liu et al. discussed the findings in their study on the potential roles of the miR-148 family in immune homeostasis and regulation. When they examined dendritic cells (DCs) stimulated by TLR agonists, they discovered that the miRNA was overexpressed upon activation, which inhibited cytokine production, upregulation of MHC class II expression, and DC-mediated T-cell proliferation. They also observed that miR-148/152 targets calcium/calmodulin-dependent protein kinase II, which could increase production of proinflammatory cytokines in response to DC activation by TLR in mice [52]. Farid et al. were able to show that the miR-122 in the serum of patients with acute rejection reaches a high level even at the beginning of rejection in comparison with the classical aminotransferase marker and may perhaps provide a new marker for early diagnosis and faster intervention during an acute liver rejection [16]. The significance of miRNA in liver transplant rejection has also been supported by animal studies conducted by Morita et al. They investigated the miRNAs involved in acute rejection of liver allografts in mice and found that miR-146a, 15b, 223, 23a, 27a, 34a, and 451 were significantly increased in the grafts while miR-101a, 101b, and 148a decreased in their expression levels [53]. Several studies have quantified circulating miRNAs as potential biomarkers for hepatitis B (HBV) and hepatitis C (HCV) infections and identified four that are differentially expressed between HBV and control groups (let-7c, miR-23b, miR-122, and miR-150) [19, 54]. HCV infection studies found a significant increase of miR-21 [55–57], and this finding may be translated into transplant studies as well. In summary, miR-122 in combination with other miRNAs seems to be a good diagnostic marker for rejection.

As a novel diagnostic marker miRNAs in exosomes can be a sensor for injury and inflammation. Bala et al. studied several miRNAs packed in exosomes of liver disease patients and they were able to identify a specific pattern of miRNA reflecting liver injury [58].

*Lung*. Guo et al. found tissue-specific miR-126 to be a lung marker [59]. Zhang et al. identified miR-126 after lung transplantation when they analyzed peripheral blood mononuclear cells [22]. In mice, it has been reported that miR-126 is highly expressed by plasmacytoid DCs and it regulates the survival and function of these cells. Moreover, this miRNA was shown to regulate production of type 1 Interferon by controlling expression of KDR that encodes VEGF-receptor 2 [60]. It is unclear if this is a contamination of lung cells during the preparation or if this is due to the fact that lung tissue is damaged after lung transplantation. There is a huge variety among the detected miRNA profiles during lung rejection and even studies using the same parameters such as DSA+ and/or BOS+ end up with no significant overlap in the miRNA profiles. Further investigation is needed to define a specific marker for lung injury.

*Renal*. The greatest number of miRNA and transplant studies is done in renal transplantations. The kidney specific miRNA-146a seems to be a risk factor in development of rejection because as Misra et al. were able to demonstrate, the mutation in miR-146 (SNP) is associated with double the risk for rejection [26]. MiRNA-146a expression is highly elevated in response to inflammatory stimuli such as cytokines. Specifically, a recent study on miRNA expression in human activated CD8+ T-cells showed that when these cells were treated with IL-2 or IL-15, miR-146a was significantly upregulated. Also, they were able to demonstrate that subsets of CD8+ T-cells, including naïve and memory cells, differentially expressed certain miRNAs, with 146a being strongly upregulated in the memory T-cell subset [61, 62]. MiR-10b is another kidney specific miRNA that regulates the expression of BCL2L11. Downregulation of miR-10b directly depressed the expression of BCL2L11. Transfecting miR-10b into human renal glomerular endothelial cells recapitulated key features of acute allograft rejection, including endothelial cell apoptosis, release of pro/inflammatory cytokines (IL-6, TNF-alpha, IFN-gamma, and CCL2) and chemotaxis of macrophages, whereas transfection of miR-10b mimics had the opposite effects [28]. Members of the miR-10 cluster were also found in other organ transplantation studies. Despite the numerous studies in kidney transplantation, there is no common marker for acute rejection and further investigation to find a marker for clinical use is necessary. Sui et al. investigated mechanisms that are related to rejection by integrating protein, mRNA, miRNA, and lncRNA in biopsies of patients with acute rejection versus controls. They were able to predict five transcription factors that are active and responsible for rejection and these transcription factors correlate with 12 miRNAs and 32 lncRNAs. In a previous study, the same author was able to confirm and validate two additional miRNAs (miR-320 and miR-324) by quantitative polymerase chain reaction [24].

*Cardiac*. Many studies describe miRNA-142-3p as an expression marker of organ grafts. It is associated with lymphocyte alloimmunity during rejection and organ damage. This miRNA is found as an overlap in the three presented studies of heart transplantation [32, 34, 35]. Van Huyen et al. identified and validated a set of four miRNAs (miR-10a, miR-31, miR-92a, and miR-155) as a specific signature for cardiac rejection. The correlation in the receiver operating curves showed a very strong and significant relationship between these miRNAs and rejection [35]. In different studies, miR-10b has been shown as an inhibitor of NFkB signaling and as a regulator of the proinflammatory markers MCP-1, IL-6, IL-8, IL-1, and VCAM [63]. For miRNA-155, Liod et al. described several inflammatory functions that include its enhanced expression after activation of the T-cell receptor, the repression of the IFN receptor, and the contribution to Ig class switch in B cells. Increased expression of this miRNA molecule is also associated with activation of DCs. MiRNA-155 is believed to possess the ability to modulate the antigen presentation activity of DCs to activate T-cells according to animal studies using a mouse model [64]. In DCs derived from human monocytes, following activation by lipopolysaccharides, the expression of miRNA-155 was upregulated as high as 50-fold. In the same study, the knockout of miR-155 in the activated DCs led to an increase in some cytokine gene expression, suggesting its potential role as a negative regulator of cytokine production [65]. Moreover, miR-155 has been found to be upregulated in graft infiltrating lymphocytes, T-cells in spleen, and circulating lymphocytes during acute cardiac rejection in mice. GSK3*β* was identified to be a direct target of miR-155, which decreased GSK3*β* expression and thereby increased proliferation of T-cells [66]. MiRNA-31 mainly regulates the expression of E-selectin and ICAM-1 when it is induced by the TNF pathway. It also regulates the infiltration of immune cells into the tissue. Finally, miR-92 targets integrin *α*5, S1P1, MKK4, and eNOS and plays a potentially important role in the vascular inflammatory response. Dawi et al. discovered three miRNAs, miR-326, miR-142-3p, and miR-101, which have crucial functions in the regulation and maintenance of self-tolerance. Further investigation of miR-142-3p in combination with an miRNA that shows organ damage could prove to be a good candidate biomarker for ongoing rejection.

More systematical research is needed to determine whether miRNAs can be applied as biomarkers, therapeutic targets, or therapeutic agents for specific organs. For other inflammatory diseases like atherosclerosis, a full network between miRNA, organs, and immune cells are described and can help to understand the function in an ongoing inflammation that can lead to chronic rejection [67]. As additional information, a great overview about organ-specific miRNAs as a potential profile for allo- and xenotransplantation is given in the review from Zhou et al. [68].

### 2.3. miRNAs as a Tool to Enhance Transplantation

The last study we would like to discuss in this review is a study that was done in hematopoietic stem cell (HSC) transplantation and that shows the positive effect of miRNAs packaged in extracellular vesicles on HSC transplantation. De Luca et al. identified miRNAs and piRNA derived from extracellular vesicles (EVs) secreted by mesenchymal stem cells. The group found 87 miRNAs and five piRNAs differentially expressed and predicted to regulate cell differentiation and apoptosis. Validation of four miRNAs and further experiments showed that the EV treatment of HSCs enhances host engraftment and HSC plasticity and function. These findings create a new perspective on how miRNA and piRNA in EVs can positively influence the outcome of transplantations [37].

In addition to this study several publications are showing a specific regulatory function of exosomes and the proteins and miRNAs transmitted by them. As an example Song et al. were able to show that donor-derived peripheral exosomes have the potential to inhibit immune inflammation in allograft heart transplantation by the specific induction of Treg cells [69]. For microvesicle-delivered miRNAs derived from endothelial progenitor cells, it was demonstrated that they are able to reprogram residential renal cells and protect the kidney from ischemia-reperfusion injury [70]. The review from Monguió-Tortajada et al. gives a great overview of the variety of these regulatory functions, from classical immunosuppression to novel extracellular vesicles [71].

## 3. Conclusion

MiRNAs are emerging as important regulatory molecules of gene expression. They play a significant role in many physiological and pathological processes and have revolutionized cellular biology in the past decade. Research has focused on different expression profiles in health and disease. In the field of transplantation, several miRNAs have been described and it has been shown that miRs have the potential to be a novel diagnostic marker. Therefore, they represent a group of promising candidates for early detection of organ rejection with the potential to affect clinical decision making. However, further investigation and standardization in the profiling of miRNAs in serum, plasma, and urine samples are needed to find a robust diagnostic marker and to develop insights into pathways responsible for the rejection process as well as novel targets for therapy. A few miRNA mimics (miR-34 in phase I) and miRNA inhibitors (anti-miR-122 in phase II) are already in their first clinical trials and show promising results in HCV and primary liver cancer treatment [72, 73]. Today we are on the verge of implementing many of these new technologies into the clinical routine to improve diagnosis and treatment of transplant patients and to enhance their quality of life.

## Figures and Tables

**Table 1:** Categories, characteristics, and functions of selected ncRNAs.

	Length	Function
*Short ncRNAs*		
miRNA	~22 nt	Posttranscriptional regulation of gene expression
siRNA	~21 nt	Gene silencing
piRNA	~30 nt	Transposon regulation, development

*Long ncRNAs*		
rRNA	+1.9 kbases	Protein biosynthesis
lncRNA	~200 nt	Epigenetics and gene regulation

**Table 2:** Human miRNA expression in different types of transplantation.

	MicroRNA	lncRNA and piRNA	Target	Source	Method	Groups	Ref.
Liver	miR-122, miR-148a, miR-194	N/A	N/A	Biopsy Serum	TaqMan miRNA assay	AR (*n* = 13) Control (*n* = 12) Total LivertTx (*n* = 43)	[16]
	miR-122, miR-155	N/A	BAAT, STAT-1	Biopsy	qPCR	RHC (*n* = 17) AR (*n* = 12)	[17]
	**miR-146a, miR-19a, miR-20a**, miR-200a, miR-141, miR-203, miR-20b, miR-205, miR-33a, let-7e, miR-150, miR-34c-5p, miR-342-5p, miR-181c-5p, miR-29a, miR-19a, miR-204, miR-20a, miR-328, miR-1336, miR-223, miR-210, miR-503	N/A	TGF*β*R2, SMAD4, EGFR, VGFA, IL-8, IL-6, CCL8, CD40L, IRS2, TLR4, c-Myb, STAT-1, IGF1, VGFA	Biopsy	GeneChip 2.0 Affymetrix and qPCR	Slow fibrosis progressors (*n* = 11) Fast progressors (*n* = 9) AR (*n* = 5) Control (*n* = 4)	[18]
	let-7c, miR-23b, miR-122, miR-150	N/A	N/A	Serum	TaqMan miRNA Assay	OBI (*n* = 11) HBV (*n* = 29) Control (*n* = 30)	[19]

Lung	miR-144	N/A	TGIF1 (TGF beta signaling)	Biopsy/BAL cells	TaqMan miRNA assay	BOS+ (*n* = 20) BOS− (*n* = 19)	[20]
	let-7c,** miR-10a**, **miR-98**, miR-99a, miR-101, miR-125a-5p, miR-125b, miR-135b, miR-137, miR-148b, miR-184, miR-190, miR-199b, miR-203, miR-219, miR-299-5p, miR-302b, miR-335, miR-338-3p, **miR-369-5p**, miR-381, miR-450a, miR-499, **miR-518f**, miR-548c-5p, miR-551b, miR-627, miR-708, miR-874, miR-208, miR-875-5p, miR-302c^*∗*^, miR-18b, **miR-133b**, miR-134, miR-145, miR-187, miR-214, miR-433, miR-489, miR-494, miR-503, miR-542-5p, **miR-548d**, miR-628-5p, miR-144^*∗*^	N/A	TGF beta and B- cell receptor signaling	BAL cells/PBMCs	TaqMan low-density array, TaqMan miRNA assay	DSA+BOS− (*n* = 10) DSA+BOS+ (*n* = 10) DSA−BOS− (*n* = 10)	[21]
	364 differentially expressed, **miR-299-3p, miR-29b-1**^**∗**^**, miR-34a, miR-451, miR-519e, miR-629, miR-590-5p, miR-381, miR-374a, miR-28-5p, miR-126, miR-27b**	N/A	TCF4, LRRC8B, C14orf2, FUT8, C14orf135, ATR, PYHINI, TCF4, CA1, NFKBIA, NFIL3, DOCK4, PLK2	PBMCs	Exiqon miRCURY LNA array	LTx (*n* = 18) Control (*n* = 35)	[22]
	miR-16, miR-195	N/A	Rfx5 MHC CII	BAL cells	TaqMan miRNA assay, qPCR	DSA+ (*n* = 15) DSA−BOS− (*n* = 15)	[23]

Renal	miR-658, miR-629, miR-628, miR-602, miR-381, miR-125a, miR-663, miR-654, miR-611, miR-524, miR-483, miR-346, miR-326, miR-324, miR-125b-2, miR-125b-1	NR_001562, NR_002791, NR_002909, NR_002941, NR_003024, NR_003130, NR_003573, NR_023318, NR_024080, NR_024332, NR_024400, NR_024418, NR_024611, NR_026550, NR_026576, NR_026695, NR_027303, uc001pyd, uc002nyb, uc002zic, uc002zpx, uc003akf, uc003bgk, uc003dwf, uc003syy, uc003tsq, uc003wcs, uc003zfx, uc010akv, uc010gqe, uc010kwo, uc010lqx	AP-1, AP-4, STATx, c-Myc, p53	Biopsy	Exiqon MiRNA microarray, LncRNA expression microarray	AR (*n* = 3) Control (*n* = 3)	[24]
	**miR-99b, miR-23b, let-7b-5p, miR-30a, miR-145**	N/A	N/A	Biopsy	NanoString assay, TaqMan miRNA assay	APN (*n* = 11), AR (*n* = 5) Control (*n* = 4)	[25]
	miR-146a C>G (rs2910164), miR-149 T>C (rs2292832), miR-196a2 (rs11614913), miR-499a A>G (rs3746444)	N/A	N/A but twofold increased risk for overall survival	Biopsy	Genomic DNA sequencing	ESRD: AR (*n* = 52) Non-AR (*n* = 218)Control (*n* = 350)	[26]
	**miR-142-5p, miR-155, miR-223, miR-30a-3p, miR-10b, let-7c**	N/A	CD3, NKCC-2	Biopsy (PBMCs/HRECs)	TaqMan low-density array, qPCR analysis	AR (*n* = 12) Normal (*n* = 21)	[27]
	miR-10b	N/A	BCL2L11	Biopsy	Next-generation sequencing, qRT-PCR	AR (*n* = 15) Normal (*n* = 15)	[28]
	**miR-99a**, miR-100, miR-151a, let-7a, let-7c, let-7f	N/A	N/A	Serum	TaqMan miRNA Assay	AR (*n* = 12) Control (*n* = 11) DGF (*n* = 15)	[29]
	**miR-324-3p**, miR-611, miR-654, miR-330_MM1, miR-524^*∗*^, miR-17-3p_MM1, miR-483, miR-663, miR-516-5p, miR-326, miR-197_MM2, miR-346, miR-658, miR-125a_MM1, **miR-320**, miR-381, miR-628, miR-602, miR-629, miR-125a	N/A	RIMBP2, GTDC1, NTRK2, CCDC21, SAMD4B, SYS1, SYNGR1, PTPN9, ETF1, BCL6, ACBR2B, NFIB, RAB11FIP2, ARID4B, PTCH1, HD, HABP4, FOXG1B, H1F0, EDEM1, ZNF673	Biopsy	Exiqon MiRNA microarray	AR (*n* = 3), Control (*n* = 3)	[30]
	**miR-182-5p, miR-21-3p**	N/A	FOXO1, BCL2	Biopsy	GeneChip miRNA 3.0, qPCR	DSA+ AR (*n* = 16) AKI (*n* = 8) Control (*n* = 10)	[31]
	miR-450b-5p, **miR-142-3p**, miR-876-3p, miR-106b, miR-508-3p, miR-148b, miR-324-5p, miR-98	N/A	TGF*β*-1	PBMCs, B-cells	TLDA microRNA cards pool A, TaqMan qPCR	Operationally tolerant (*n* = 15) STA (*n* = 20)	[32]
	**miR-10a, miR-10b, miR-210**	N/A	N/A	Urine	TaqMan qPCR	Acute cellular rejection (*n* = 62) Control (*n* = 19)	[33]
	**miR-25, miR-181a, miR-204, miR-192, miR-10b, miR-142-3p, miR-215, miR-342-3p, miR-615-3p**	N/A	PRMT5, TP53, CDX2, ATM, HIPK2, TGF*β*R1, TGF*β*R2, SNAI1, SPDEF, MAD2L1, HRH1, LMNB2, DTL, NCOR2, RAC1, ACVR2B	Biopsy	Whole genome microarrays, microfluidic qPCR	AR (*n* = 18) Control (*n* = 52)	[34]

Cardiac	**miR-10a, miR-21, miR-31, miR-92a, miR-142-3p, miR-155, miR-451**	N/A	Inhibition of NFkB signaling pathway, inflammatory pathways E-selectin, ICAM-1, *α*5, S1P1, MKK4, eNOS	Serum	qPCR analysis	AR (*n* = 30) Control (*n* = 30)	[35]
	miR-326, miR-142-3p, miR-101, miR-144, miR-27a, miR-424, miR-339-3p	N/A	N/A	Serum	qPCR analysis	Before AR (*n* = 10) During AR (*n* = 10) After AR (*n* = 10)	[36]

BM	**miR-27b-3p, miR-10a-5p, miR-21-5p, miR-181a-5p, miR-92a-3p, **miR-3168, miR-22-3p, miR-378a-3p, miR-205-5p, miR-423-5p, miR-146b-5p, miR-26a-5p, miR-148a-3p, miR-486-5p, miR-28-3p, miR-4792, let-7a-5p, miR-3182, miR-423-3p	piR_004307_DQ575881, piR_017723_DQ594464, piR_020814_DQ598650, piR_016745_DQ593052, piR_002732_DQ573682	EGR2, CEBPA, ANXA1, MPL, ZFP36, CCL3, CSF2, IL1b, CXCR4, GATA1, MPO, SLC2A5, SLAMF8, CYP1B1	MSC and cord blood CD34+	Next-generation sequencing and qPCR	CD34+ with EVs (*n* = 3) CD34+ Control (*n* = 3) EVs (*n* = 3)	[37]

Occult HBV infection (OBI), hepatitis B virus (HBV), acute rejection (AR), sustained responders (SR), acute pyelonephritis (APN), bronchiolitis obliterans syndrome (BOS), development of antibodies to HLA (DSA), end-stage renal disease (ESRD), delayed graft function (DGF), acute tubular necrosis without rejection (AKI), stable patients treated with conventional immunosuppression (STA), extracellular vesicle (EV), recurrent hepatitis C (RHC), peripheral blood mononuclear cell (PBMC), bronchoalveolar lavage (BAL), human renal epithelial cell (HRECs), and bone marrow (BM) bold = second validation by quantitative polymerase chain reaction. *∗* = star strand of the miRNA duplex (less predominant loaded to RISC).
